# Perspectives on deployment of humanitarian workers through operational partnerships during the acute emergency health response to the Rohingya refugee crisis in Cox’s Bazar

**DOI:** 10.1186/s12873-022-00618-4

**Published:** 2022-04-07

**Authors:** Claire Blackmore, Egmond Samir Evers, S. M. Asif Sazed, Amrish Baidjoe, Victor Del Rio Vilas, Art Pesigan, Roderico Ofrin

**Affiliations:** 1 World Health Organisation, Regional Office for South-East Asia, World Health House, Indraprastha Estate, Mahatma Gandhi Marg, New Delhi, 110 002 India; 2 World Health Organisation, Cox’s Bazar Emergency Sub-Office, Sea Palace Hotel, Kolatoli Road, Cox’s Bazar, 4700 Bangladesh; 3 Global Outbreak Alert and Response Network (GOARN), WHE/EMO, Office E225, Avenue Appia 20, 1211 Geneva 27, Switzerland; 4 London School of Hygiene and Tropical Medicine, Department of Infectious Disease Epidemiology, London School of Hygiene & Tropical Medicine, Keppel Street, London, WC1E 7HT UK

**Keywords:** Humanitarian response, Health emergencies, Operational deployments, Refugee setting, Human resources for health

## Abstract

**Background:**

The unprecedented influx of Rohingya refugees into Cox’s Bazar, Bangladesh, in 2017 led to a humanitarian emergency requiring large numbers of humanitarian workers to be deployed to the region. The World Health Organization (WHO) contributed to this effort through well-established deployment mechanisms: the Global Outbreak Alert and Response Network (GOARN) and the Standby Partnerships (SBP). The study captures the views and experiences of those humanitarian workers deployed by WHO through operational partnerships between December 2017 and February 2019 with the purpose of identifying challenges and good practice during the deployment process, and steps to their improvement.

**Methods:**

A mixed methods design was used. A desktop review was conducted to describe the demographics of the humanitarian workers deployed to Cox’s Bazar and the work that was undertaken. Interviews were conducted with a subset of the respondents to elicit their views relating to their experiences of working as part of the humanitarian response. Thematic analysis was used to identify key themes.

**Results:**

We identified sixty-five deployments during the study period. Respondents’ previous experience ranged between 3 and 28 years (mean 9.7 years). The duration of deployment ranged from 8 to 278 days (mean 67 days) and there was a higher representation of workers from Western Pacific and European regions. Forty-one interviews were conducted with people who experienced differing aspects of the deployment process. Key themes elicited from interviews related to staffing, the deployment process, the office environment and capacity building. Various issues raised have since been addressed, including the establishment of a sub-office structure, introduction of online training prior to deployment, and a staff wellbeing committee.

**Conclusions:**

This study identified successes and areas for improvement for deployments during emergencies. The themes and subthemes elicited can be used to inform policy and practice changes, as well as the development of performance indicators. Common findings between this study and previous literature indicate the pivotal role of staff deployments through partnership agreements during health emergency response operations and a need for continuous improvements of processes to ensure maximum effectiveness.

**Supplementary Information:**

The online version contains supplementary material available at 10.1186/s12873-022-00618-4.

## Background

In 2017, over 700,000 Rohingya refugees crossed from Myanmar into Bangladesh joining refugees from previous waves of displacement [1–3] to form the largest refugee camp in the world, currently hosting over 850,000 refugees, and straining the resources of one of Bangladesh’s poorest districts [4].

The Rohingya refugees faced considerable public health challenges, including severe and acute (mental) trauma, housing in overcrowded camps, poor water sanitation and hygiene (WASH) conditions, poor nutritional status [5], low vaccination coverage [6] and others.

The number of refugees rapidly overwhelmed the limited available capacity of local communities and health systems, and additional health service capacity was required. The Government of Bangladesh requested international assistance, and the World Health Organization (WHO) took on the leadership of the Health Sector Coordination in support of the Government on 1st October 2017. In an emergency of this scale, it was necessary for WHO to collaborate with operational partners in line with the Regional Framework on operational partnerships for emergency response to achieve a timely, coordinated, effective and efficient emergency response [7]. Standby Partnerships (SBPs) and the Global Outbreak Alert and Response Network (GOARN) contributed significantly to the staffing of WHO operations in Cox’s Bazar.

Standby Partnerships are bilateral agreements between organizations and UN agencies, with partners providing support to UN agencies responding to humanitarian emergencies through the secondment of gratis personnel. Each Standby Partner maintains its own roster of humanitarian experts [8]. These personnel have been pre-identified and can be rapidly mobilized.

GOARN is a global partnership which can deploy personnel with technical and operational skillsets to support public health emergency response [9]. The GOARN secretariat and operational support team are based in WHO Headquarters in Geneva, Switzerland, and at several WHO regional offices. Partners include networks of academic institutions, laboratories and regional technical networks, medical and surveillance initiatives, United Nations agencies, international humanitarian non-governmental organizations and national public health institutions.

### Previous research into deployments

Humanitarian emergency response globally involves the deployment of large numbers of staff, often at significant financial cost [10]. Research is needed to guide organisations to undertake these deployments efficiently, while ensuring the thorough vetting and wellbeing of deployed staff.

Limited literature has been published on staff providing humanitarian assistance [11]. Where it is available, the majority has been concentrated on the mental wellbeing of humanitarian workers, or focuses specifically on recruitment or the nature of the work. In addition to the operational constraints of research in humanitarian settings, workers deployed are often part of a transient workforce for which follow up can be challenging [12]. To date, there has been little written on the process of their deployment, or the technical and operational aspects of the deployment process in the emergency response.

Work was initiated by WHO in order to analyse and evaluate the deployment processes and experiences in the emergency response in Cox’s Bazar. This article aims to describe the demographic characteristics of deployees through operational partnerships mechanisms, and to describe deployment experiences from personnel within different roles to identify challenges and good practice during the deployment process, and how the process could be improved. Results of this study can inform future changes in policy and practice related to deployments in humanitarian emergencies.

## Methods

A mixed methods approach was used: firstly, a desk review of deployments utilizing quantitative data was performed, followed by a qualitative study of interviews carried out with personnel involved in the response by operational partnerships in Cox’s Bazar.

### Desk review

#### Study design

The desk review was a cross-sectional study from which descriptive statistics were derived to characterize the demographics and input of people deployed by GOARN and SBPs in Cox’s Bazar.

#### Study population

The study population comprised of people deployed to Cox’s Bazar through either GOARN or a Standby Partner as part of the emergency response to the Rohingya refugee crisis between December 2017 and February 2019.

#### Data collection

Data were extracted from a database held centrally at WHO Headquarters in Geneva, which included demographic details of deployees, dates of deployment, deployee role (job title) and incident management system function (wider technical area of working), releasing entity (operational partner) and grade. Previous experience and nationality were extracted by the authors (EE,CB) from CVs submitted to WHO as part of the application process. Descriptions of contributions were extracted by the authors (EE,CB) from technical reports and performance evaluation reports submitted by the workers at the end of their deployment to provide an overview of the activities undertaken and aid interpretation of the data. All information was collated into a core dataset in Excel, from which descriptive statistical analysis was performed (CB). Where data were missing, alternative data sources were used to attempt to complete the dataset where possible. The number of deployees included in each statistical analysis is indicated in the relevant results section. This dataset was also used as a reference to interrogate the qualitative data, looking for patterns in responses from particular technical areas or organisations.

### Interviews of personnel

#### Study population

Interviewees were selected persons with significant operational experience related to the deployment of operational partnership personnel, where their activities linked to the deployment process was a substantial proportion of their role. This included WHO staff from all levels of the organization and staff from operational partners who contributed to the deployment process. Deployees of all experience levels were included in the interviews, with some having had several previous deployments to other locations and some having had no previous deployments. Inclusion criteria are included in Supplementary Materials.

#### Study design

A semi structured interview with a set of core questions was used, with flexibility to probe interviewees’ answers further if needed. Interview questions were developed by the authors (AP, EE, CB) and designed to cover key elements of the operational partnership response. Questions used are included in Supplementary Materials, and covered the interviewees’ role, their view on the contribution of operational partnerships in Cox’s Bazar, challenges faced and suggested improvements.

#### Data collection

The interviews were conducted by an external consultant (CB), unknown to the majority of interviewees. The setting of the interview varied: ideally interviews were conducted face to face, but where this was not practical, online or telephone interviews were used.

#### Sampling technique

Initially a purposive and stratified sampling frame was used (AP and EE), identifying twenty people representing each technical area or role of interest. This included at least one representative from each area listed in Table 1 and their affiliation or location, listed in Table 2. As interviews progressed, a snowball sampling technique developed, with interviewees suggesting others to interview. Interviews were conducted until suggested interviewees had responded and saturation point was reached and subsequent interviews offered no new insights.

**Table 1:** Job roles and related IMS (Incident Management System) function for staff deployed to Cox’s Bazar December 2017 – February 2019

Deployee IMS function	Deployee role	Number of deployments (%)
Health expertise and operations	Case management officer	3 (4.6)
	Epidemiologist	18 (27.7)
	Field manager	4 (6.2)
	Health operations team lead	1 (1.5)
	Infection prevention and control	6 (9.2)
	Laboratory technical officer	2 (3.1)
	Public health officer	1 (1.5)
	Surveillance officer	9 (13.8)
	Epidemiology team lead	1 (1.5)
	Mental health technical officer	1 (1.5)
	WASH officer	4 (6.2)
Leadership	Communications officer	3 (4.6)
	Resource mobilization officer	1 (1.5)
Operations support and logistics	Health logistics officer	1 (1.5)
	Operations support and logistics team lead	1 (1.5)
Partner coordination	Health cluster coordinator	1 (1.5)
Planning and information	Data management officer	4 (6.2)
	GIS specialist	1 (1.5)
	Information management team	3 (4.6)

**Table 2:** Demographic information on deployees to Cox’s Bazar, as of 1 February 2019 (total 65 deployments)

Characteristic	Number of deployees (%)
Gender	
Female	33 (50.8)
Male	32 (49.2)
Missing data	0 (0)
Nationality (by WHO Region)	
Europe	26 (40.0)
Western Pacific	14 (21.5)
Pan America	11 (16.9)
Eastern Mediterranean	6 (9.2)
Africa	5 (7.7)
South-East Asia	1 (1.5)
Missing data	2 (3.1)
Age (years)	
20-29	2 (3.1)
30-39	9 (13.8)
40-49	11 (16.9)
50-59	5 (7.7)
60+	1 (1.5)
Missing data	37 (56.9)
Years of experience	
0-5	18 (27.7)
6-10	26 (40.0)
11-15	11 (16.9)
16-20	7 (10.8)
21-25	2 (3.1)
26+	1 (1.5)
Missing data	0 (0)
Highest educational attainment	
Bachelors degree	5 (7.7)
Masters degree	41 (63.1)
MD	8 (12.3)
PhD	10 (15.4)
Missing data	1 (1.5)

#### Analysis

Thematic analysis of the interviews was undertaken (CB) and followed the seven steps outlined by Braun and Clarke: transcription, reading and familiarization, coding, searching for themes, reviewing themes, defining and naming themes, and finalizing the analysis [13]. Discussions were held by members of the authorship team (CB, AP, AB) to clarify and triangulate themes and gain a fuller understanding of the interviewees’ discourse. Qualitative data were interpreted and analysed in conjunction with the quantitative analysis, allowing interview responses to be informed by, and interpreted in light of the related quantitative data.

#### Ethical approval

As this study is a service evaluation and no patients were involved, there was no requirement for ethical approval. Involvement in interviews was optional with no remuneration, and participants were informed verbally of the aims of the study at the start of the interview by the interviewer. It was discussed that the purpose was to conduct an internal review the operational response, and the interview was designed and would be conducted by WHO. Participants were informed they were free to withdraw from the process at any point and their responses would be removed. They were also informed that information provided would be included in the review, but any statements used would be anonymised. The interview did not proceed until interviewees verbalised that they understood and agreed to participate.

## Results

### Desk review

#### Deployments of personnel

There was a total of 65 deployments of personnel from operational partners to the WHO Cox’s Bazar emergency office between December 2017 and February 2019: 43 deployments through GOARN and 22 through SBPs. Four people were deployed twice, two from GOARN and two from SBPs, giving a total of 61 individuals who were deployed during that period.

The majority of deployees fulfilled roles within the health expertise and operations function of the Incident Management System (IMS), as outlined in the WHO Emergency Response Framework [14], with the largest number of deployees fulfilling epidemiologist or surveillance officer roles. Specific job roles are included in Table 1.

### Demographic information on deployees

Available demographic information on the deployees is displayed in Table 2. Of the 65 deployments, 32 were male and 33 were female. Two women and two men were deployed twice. Information on nationality was available for 63 of the 65 deployments. The highest number (26 deployees, 41.2%) came from the EURO region.. One deployee (1.6%) came from within the South-East Asia region (where Cox’s Bazar is located). Deployees’ relevant occupational experiences were classified as those where the job title or role description on the CV was related to the role they were deployed to fulfil. This was not always directly linked to humanitarian or the public health sector, with some deployees having relevant experience from the private sector. The number of years of experience ranged from three to 28 (mean 9.7). All deployees except one provided educational achievements on their CV, with all being in possession of at least a Bachelor’s degree.

### Duration of deployment

**Fig. 1:**
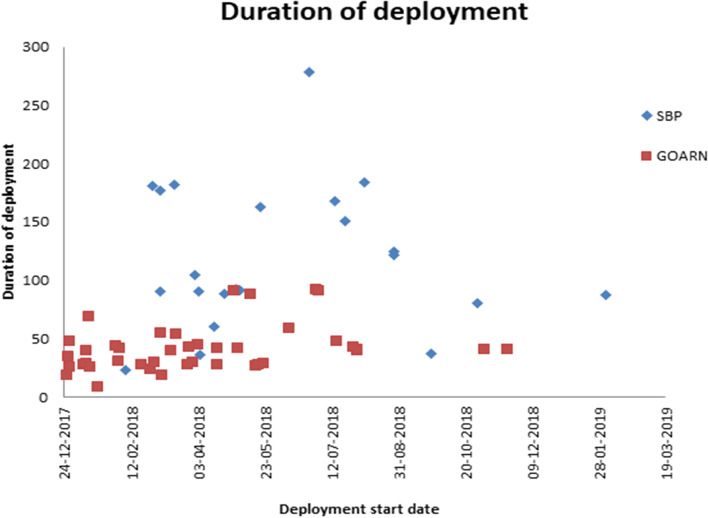
Duration of deployment (in days) of deployees within the WHO Cox’s Bazar emergency office by deployment mechanism, December 2017 - February 2019

**Fig. 2:**
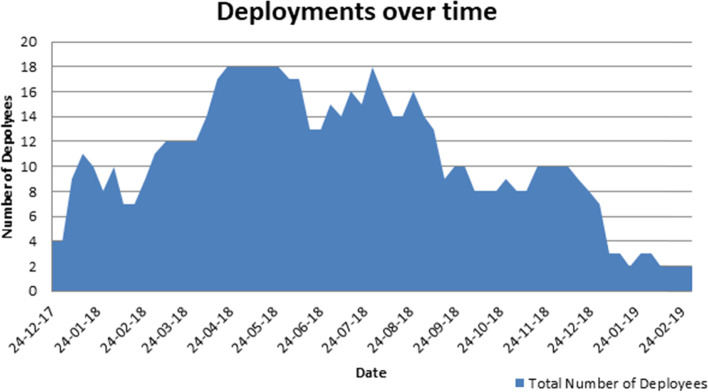
Number of deployees within the WHO Cox’s Bazar emergency office December 2017 - February 2019

The length of deployment ranged from eight days to 278 days, shown in Fig. 1. The mean length of deployment was 67 days, with GOARN deployments tending to be shorter (mean 40 days, range 8-91) and deployments through SBPs lasting longer (mean 119 days, range 23-278). The longest deployments were between March and August 2018. This is also reflected when looking at the number of deployees in the Cox’s Bazar office at any given time (Fig. 2). This peaked at 18 deployees in May and July 2018, then reduced gradually over time.

### Interviews of personnel

Forty-one interviews were conducted with people involved in the deployments of personnel to Cox’s Bazar. Their affiliation is shown in Table 3. This includes 11 of the deployees from different job roles in Table 1, and 30 personnel (both WHO and external to WHO) who were either involved in the deployment process or worked directly with the 65 people described above. The majority of interviews took around 1 h to complete.

**Table 3:** Location of interviewees

Group, by location	Number of people
WHO Headquarters (Geneva, Switzerland) including GOARN and SBP secretariats	4
WHO South-East Asia Regional Office (New Delhi, India)	7
WHO Bangladesh Country Office (Dhaka, Bangladesh)	5
WHO Cox’s Bazar Emergency Office (Cox’s Bazar, Bangladesh)	11
Deployees	11 (SBP = 6, GOARN = 5)
SBPs: representatives from RedR Australia, Save the Children UK and Norwegian Refugee Council	3

*SBP* Standby partner

### Key themes

Four key themes were elicited by thematic analysis of the interviews: “staffing”, “deployment process”, “office” and “capacity building”. Within these four key themes, there are between two and four master themes, and several subthemes. These are shown in Table 4, with example quotes where interviewees suggested areas for development for each subtheme.

**Table 4:** Themes and subthemes identified from interviews with example quotes

Themes	Master themes	Subthemes	Example quotes
1. Staffing	Team structure	Incident Management Structure (IMS)	“I did not feel it was a hierarchical environment” “There are communication gaps within the IMS, both horizontally and vertically” “Some people from partnerships are trained and well versed in how WHO works and the IMS”
		Lead roles/team positions	“Leadership should be taken on by more experienced deployees… if they are here long enough” “It is better to give specific technical functions to deployees, rather than lead positions or management responsibility, as these roles should be given to people who are present for a longer period of time”
	Continuity and transition	Duration of deployment	“There should be a minimum deployment length of three months” “The same time is spent on recruitment regardless of how long they stay for, but the deliverables are very different” “Those who contributed the most were those who stayed for more than 3 months” “GOARN deployees are usually only for six weeks, it would be good if this could be extended”
		Long-term staffing plans	“[Operational partnerships] should be used [only] for deployment acutely” “We are moving towards recruiting longer term staff, especially national staff. It would work well if experienced deployees train the national staff”
	Role of deployees	Terms of reference (ToR)	“Terms of reference need to be clear, both for the receiving office and the deployee” “[unclear terms of reference] results in time not being properly utilized” “Terms of reference are very broad.” “Contributions were greater when there was a clear ToR with tasks that could be achieved in the short space of time”.
		Handover	“Deployments should be staggered, with no gaps” “There should always be handovers”
		Debriefing	“There should be a debriefing at the end of each deployment with a focal point from the organization” “[Deployees] would find ways of handing over what was left and gave ideas on how it could be continued. They were mature, highly qualified, and concerned about handover”
	Quality of deployee performance	Performance Evaluation Report (PER)	“(The PER) is a very rigid evaluation structure [it] needs to include softer skills as well as the technical skills” “An alternative to the PER could be a structured conversation” “There is a need for an internal record that is distinct from the PER”
		Roster	“A roster should be formed of individuals who have been to CXB and performed well, who we could ask to return”
		Performance review	“There should be a performance review process and an early evaluation” “More feedback to deployees and deploying organizations is needed” “An early assessment should be undertaken after two weeks”
2. Deployment process	Preparation and arrival	Preparation	“Before initiating the deployment, you should receive letters, documents, and in good time… you need some internal documents and information” “Deployees are usually informed about procedures for payment, leave etc by their deploying organization before leaving, so usually this is straightforward” “Personnel deployed through GOARN have been briefed by GOARN, and there is a GOARN focal point”
		SBP/GOARN deployment	“Deployments from GOARN/SBP were a life-saver when we needed one” “Deployments need to be more timely and reliable” “GOARN personnel deployed were highly trained and deployed quickly”
		Organizational structure	“Administrative questions at times must pass through 3-4 layers: field office administration, country-office, regional office and at times headquarters/global service center… which introduces a delay”
		Training	“Training needs to be provided on the systems required” “Different SBPs may offer different training, but all will receive a degree of mandatory training on operations, finance, security” “In every position there are some particular training needs”
		Orientation/briefing	“It would be good to have a briefing for deployed personnel in a more structured way”
	Recruitment	SBP/GOARN recruitment and selection	“Technical area experts should advise the GOARN/SBP partners to recruit deployees/technical experts with the required skillsets” “Generally there has been appropriate matching between expertise, experience and local context” “[An] advantage of using SBPs to deploy staff is that often staff know each other through being deployed with the same people previously”
		Telephone call/Skype	“Although deployees are pre-selected through rosters, would still recommend having an interview or call on Skype before agreeing to deployment to discuss expectations”
3. Office	Environment	Office environment	“There were no set seating arrangements… members of the same team were at time distributed across different rooms” “We now pay more attention to staff wellbeing” “We have a staff wellbeing team trying to enhance team spirit and have everyone working as one” “The SBPs brought diversity to the office – they were from Kenya, Somalia, Afghanistan, Australia, Sudan”
		Wider environment	“Individuals can have cultural sensitivity and be adaptable but it depends on the person and their experience” “Generally, staff were experienced in working in different cultures and did not have trouble adjusting” “There are less complex security issues than in other contexts where I have worked”
	Policies and procedures	Rest and recuperation (R&R)	“A clear [standard operating procedure] is needed to ensure there is no confusion over R&R policies applicable to different contract types and situations”
		Research	“A mechanism is needed which makes it easy to do research”
		Equipment/emails	“My official email came only half-way through my deployment. Some deployees were temporarily using their personal email. Official email accounts should be assigned as a matter of routine”
	Support	Supervision	“Supervision is key for less experienced staff”
		Relationship with SBP/ institution	“There should be stronger communication between [operational partners] and WHO at field level to better meet needs”
4. Capacity building	Sharing experiences	Mentoring	“[An operational partner] has a buddy system where more junior staff are linked with seniors who have 5-10 years’ experience. This works well”
		Building collaborations	“No single institution has all the capacity and so we need to use surge capacity from other institutions”
	Increase of SEARO participation	Pool of institutions	“We want to build capacity… institutions from SE Asia are not yet as active/engaged as from other regions” “The response from Western institutions is disproportionate”
		Regional focus	“It can be useful to use regional [operational partners] due to culture and regional solidarity” “We should have a regionally focussed GOARN mechanism for this WHO region. This way the experience can be used in the region and we build local capacity” “If people are from the region or have experience in the region, they find it easier and blend in well”

#### Key theme 1: staffing

Staffing was an issue highlighted by all interviewees. All those who were interviewed felt that staff deployed through partnership agreements played a pivotal role in the emergency response.

The need for a clear team structure and reporting lines was emphasised, including communication of management decisions. Some interviewees believed the vertical structure of the Incident Management Structure (IMS) affected communication, at times preventing information from being shared both between (horizontally) and within (vertically) IMS pillars.

Some interviewees recommended that team lead positions should be assigned to persons with considerable WHO experience and who would remain in the emergency office for longer, allowing for institutional memory and more effective functioning of the IMS. This also linked with continuity, with shorter deployments viewed as having some valuable contributions but at times being disruptive. However, it was recognised that the intensive workload, especially in early stages of the response, may not be sustainable for longer periods. Many interviewees called for a longer-term staffing plan as soon as it was clear that the emergency would be prolonged.

Clear terms of reference were raised by the majority of interviewees. Some deployees suggested for these to include a degree of flexibility and to be finalized on arrival with their supervisor to ensure that they are clear on the role requirements. Handover and debriefing were also seen as important elements, and that these processes should be formalised, ideally with both the inbound and outbound deployments overlapping on site.

Many persons interviewed expressed that the standardized performance evaluation report (PER) was not seen as sensitive enough to act as an adequate evaluation tool and had limited use in distinguishing successful deployments. Linked to this, an internal roster was seen by some as a way to positively distinguish deployees who had worked well in Cox’s Bazar. Many suggested an early performance review, within the first 2 weeks of a deployment, in order to identify any potential problems and correct course as soon as possible.

#### Key theme 2: deployment process

There were requests for more information prior to deployment, both role-specific and general information about WHO systems and the WHO Emergencies programme. Preparation documents and processes varied depending on the deploying agency, with GOARN and each SBP having different guides and documents. Staff from the Cox’s Bazar emergency office commented that the timings of deployments would have the greatest added value if they coincide with greatest need and allow for handover from outgoing personnel. It was also felt that the process could be streamlined to reduce delays such as travel approvals and visas. Interviewees recognised that ideally everyone would be trained and familiar with WHO systems and procedures, but a minimum standard training in relevant processes and IT systems would be beneficial for all deployees. Alternatively, recruitment of more administrative support familiar with WHO systems could be considered. The importance of a thorough briefing was noted, specific to Cox’s Bazar and including information on context, local culture and expectations.

More transparency was requested around recruitment of deployees, including selection of candidates. It was requested by those in the emergency office that they have a more active role in the recruitment process, and for a call prior to deployment between deployee and the receiving team to improve the preparation of both parties.

#### Key theme 3: office

The temporary nature of the Cox’s Bazar emergency office premises, housed in hotel apartment blocks with several smaller rooms spread over different floors was raised. The layout was seen as contributing to fragmentation and detachment. It was raised that different office space might contribute to stronger coherence within and between teams.

Security restrictions and the cultural contextual challenges were raised, particularly by female deployees. Although considered important for staff wellbeing and productivity by all interviewed, applicable policies on leave and rest and recuperation were not clear for different contractual modalities and deployment types, resulting in perceived barriers in accessing this entitlement.

Some deployees noted that opportunities for valuable research and documentation which could improve public health practice existed in Cox’s Bazar, together with some enabling factors for research. At the same time, it was noted that some of these were missed due to operational challenges of conducting research in an emergency, as well as lengthy and unclear approval processes, questions around ownership of data and authorships. It was felt that responsibility for coordination of research efforts should be assigned within the IMS structure to a staff member based in the office for medium term.

Many deployees reported challenges with IT equipment and access, with delays in allocations of official laptops and email accounts leading to temporary use of personal devices, emails and cloud accounts. Concerns were raised around data storage, protection, and security. Establishing use of generic email addresses and function specific accounts was recommended to promote continuity, particularly for high-turnover roles.

Operational supervision and support for issues both inside and outside of work within the office were key issues, especially for less experienced deployees, although this was seen to improve with the introduction of a staff wellbeing committee later in the emergency response. The request for support also included a stronger relationship between the operational partners and WHO at field level to permit more tailored identification of requirements, as well as to address concerns regarding deployees.

#### Key theme 4: capacity building

Interviewees recognised the importance of building local capacity. Suggestions for this included a roster for personnel with appropriate skillsets for different technical functions, particularly from within the region and increasing the number of local institutions and organizations with operational partnerships in place. This regional focus was seen as important to ensure that deployees have more familiarity with local customs and culture, and be better placed to quickly form relationships and build trust with the affected populations and local staff and administration.

Mentoring was mentioned several times in relation to the need for experienced staff, with suggestions for more experienced deployees to act as mentors to allow less experienced deployees to be deployed safely. Similarly, sharing experiences was linked to building collaborations between WHO and other institutions, as well as between the institutions themselves.

## Discussion

### Demographics and professional profiles of deployees

Analysis of staff demographics showed an equal gender split, and an overrepresentation of staff recruited from Europe and Western Pacific regions. Only one deployee was from the South-East Asia region, despite interviewees stating that local deployees may have adjusted better to the environment and would have had more local context-specific knowledge. There was a large number of people deployed to work within surveillance and epidemiology, which are key technical areas during an initial emergency response when little is known about the target population and its healthcare needs [15]. Deployees generally brought considerable experience in humanitarian and public health work. There was a positive view of more experienced deployees nurturing more junior colleagues to share their skills and knowledge, and this was discussed by both deployees and permanent staff from the WHO offices. In some situations, a compromise may be made between experience and offering opportunities to newer workers to develop capacities to ensure sufficient staffing numbers, including through early recruitment of local staff and subsequent capacity building. Where this is the case, capacity building theory [16] should be considered to identify the specific theory and methodology being used to support these efforts.

### Practical considerations

Cultural challenges faced by deployees relating to the local context and the office environment were not unique to this study. Working in a markedly different cultural context was often discussed in interviews and had been mentioned in the research carried out by Bjerneld et al. [12]. This was voiced most often by female deployees and led to them feeling uncomfortable at times in the wider environment of Cox’s Bazar. Whilst this is concerning, and it has been shown that cultural contradictions between beneficiaries and agencies can impact aid effectiveness [17], both social and organisational support can offset burnout related to cultural challenges [18] and should be considered from the start of future responses to increase health and wellbeing of those deployed. Challenges relating to IT availability and access, data protection and continuity had also been raised in other studies [10, 19] and was frustrating to many of the deployees who were reluctant to use their personal email and IT equipment. Although WHO staff were not affected, all those spoken to recognised the risk of using external file sharing websites and the use of generic email addresses was recommended to provide more continuity to the response. It is recognised that the effective use of information and communication systems in emergencies can enhance the health, safety and resilience of displaced populations [20]. Where it is suboptimal, this impact is witnessed in human, organizational, and environmental issues [21] and this was seen to affect the working environment in Cox’s Bazar, where it compounded problems relating to the physical properties of the emergency office. It was important for deployees to be working in the same office area as their team but this was not always possible in the Cox’s Bazar emergency office due to the office layout. People working in the response from all technical areas who were affected by this described feeling isolated at times due to being physically distanced from team members. Physical office space arrangements in future emergencies could consider the likely size and structure of the teams necessary, and be planned to promote teamwork. This should also take into account the need for supervision and support, which was considered particularly important by less experienced staff.

### Operational considerations

The need for early performance reviews of deployees was recognised by all those interviewed and recommended to improve effectiveness and support. Other issues relating to team organisation and structure, and the need for clear and specific terms of reference, had been identified by previous research [22–24]. Those deployed felt that clarity in their roles, rather than generic terms of reference, would lead to faster orientation and integration within their teams. Likewise, requests for more information prior to deployment and a more streamlined deployment process have been found in prior studies [12, 25]. A standardised process and battery of documents could reduce these discrepancies and lead to deployees feeling equally prepared on arrival. Requests for more information on the selection and deployment of staff from operational partnerships was specific to this study and was discussed more by those who worked directly with deployees in the emergency office.

### Strategic planning

There were different opinions on optimal duration of deployment. Some interviewees felt that six-week deployments were a sufficient period of time to offer meaningful contributions within the high-pressure environment of the emergency response, while others felt that contributions made during six-week deployments often did not weigh up to the disruptive effects of staff turn-over that were experienced and advised against shorter term deployments. There was no clear pattern in preference between those deployed and permanent staff, or between those working in the emergency office or those working in other locations. However, it was recognised that longer deployments would require breaks in service for rest and recuperation. The process for this should be consistent and transparent, as requested in some interviews by those deployed who were affected by this, as it has been found to be an additional source of stress for humanitarian aid workers [26] and can disadvantage local staff [27].

Staffing levels were deliberately higher at times of predicted greater need, such as the monsoon season (see Fig. 2), and deployment length in future responses may need to be adjusted to cover times when it is possible to anticipate increased demand on services. As the response became more protracted, the reliance on surge staffing fell and there was a shift towards a more sustainable model of locally recruited staff.

### Actions taken since data collection

Since this exercise was conducted in 2019, various issues raised in this piece have been addressed, but are included here so other settings might learn from the Cox’s Bazar experience and address any issues in a timely manner. Notably in 2018, a staff wellbeing committee was established, which has since worked to flag staff wellbeing needs within the Cox’s Bazar office, to formulate advice to management and to help address wellbeing challenges where possible. Following a broad operational review in late 2018, the establishment of a sub-office structure in 2019, with delegations of authority to field level, clear administrative standard operating procedures and recruitment of administrative support staff with appropriate skillsets have been observed to have streamlined administration considerably overall. In late 2019, a move to medium-term oriented office space addressed some of the limitations flagged by deployees in the earlier office accommodation, which was perceived to limit team’s functionality. A consultant was hired specifically to coordinate and further research efforts conducted in the duty station in 2019, and GOARN has initiated an online interactive training to raise awareness among those eligible to be deployed on the realities and challenges of working with GOARN in the field on an outbreak response mission. The development of a strategic plan for the health response, structured organizational workplans and mobilization of funding are some priorities to ensure staffing can be transferred from operational partners to organizational staff as early as possible.

## Limitations

The study does not cover internal deployments within WHO, which were largely from the South-East Asia region, particularly the WHO country office in Bangladesh. It also does not cover Government deployments, including from the national FETP programme.

The selection process for interviews was originally a stratified sample but this transitioned to a snowball strategy which may have resulted in selection and reporting bias and resulting in the same issues being raised and interviews reaching saturation point earlier. The interviews, however, continued to elicit a range of both positive and negative comments on a range of topics. There are likely to also be some limitations with the transferability of findings to other groups and organizations as some issues raised may be specific to WHO policies and procedures.

Whilst it can be difficult to generalise the experience of all people deployed given their varying lengths of deployment and deployment to different areas of the response, there are common findings between this study and findings from literature which indicate continued need to improve the process of deployment of humanitarian workers for effective response.

## Conclusion

Studies have recognised a dearth of organisational learning when examining the impact of emergency work on humanitarian workers [10]. This study has examined the experiences of field workers deployed to a humanitarian emergency response and identified four main areas which contribute to the perceived quality and effectiveness of the deployments which may be considered to improve future deployments. The study has underscored the invaluable role of operational partnerships, early in emergency response, while medium-term oriented planning is being operationalized. The protracted nature of the humanitarian crisis in Cox’s Bazar has enabled this study to be designed and implemented whilst the emergency was ongoing. This allowed interviews to take place with key personnel, with many deployees still available for interview. This demonstrates feasibility of organizational operational research in a protracted emergency. The engagement of deployees in the evaluation of the response is something that must be conducted on a routine basis. It has identified themes (staffing, the deployment process, the office/base, and capacity building) which are seen as crucial to the quality and effectiveness of the deployment by both the people deployed and the receiving organisation. A number of these themes can lend themselves for policy and practice changes, as well as conversion into performance indicators for deployments for both the deploying and receiving organizations. Currently, only individual performance monitoring exists (in the form of performance reviews), but the development of wider monitoring and evaluation tools will help to assess the effectiveness of the deployments and improve their quality, for the individuals being deployed, their deploying organizations and the receiving organisation. The interviews conducted identified both strengths and areas for development in the themes identified, and it is hoped these can act as a trigger to conduct such monitoring and evaluation in future. This study provides the basis for further research into the operational and technical aspects of deployments during an emergency public health response. The aspects captured would constitute an important part of a comprehensive review of operations.

## Supplementary Information

**Additional file 1.**

## Data Availability

The datasets used and/or analysed during the current study are available from the corresponding author on reasonable request.
