# Supplementary material for: Perspectives on deployment of humanitarian workers through operational partnerships during the acute emergency health response to the Rohingya refugee crisis in Cox’s Bazar
Source: BMC Emerg Med. 2022 Apr 7;22:60. doi: 10.1186/s12873-022-00618-4 (PMC8991559; doi:10.1186/s12873-022-00618-4)
Supplement: Supplementary file 1 — Additional file 1. [file 12873_2022_618_MOESM1_ESM.docx]

# **Supplementary Materials:**

Supplementary Materials 1: Interview questions

Semi-structured questions used for interviews:

1. What has your role been relating to the CXB Rohingya emergency response?

2) In your experience, how do GOARN and SBPs contribute to the WHO response in CXB? Has this changed over the course of the time you have been in CXB?

3) What challenges are faced with regard to GOARN and SBP deployments?

4) How could GOARN and SBP deployments be improved in future, for this and future responses?

Supplementary Materials 2: Inclusion criteria

Inclusion criteria for interviewees:

- WHO staff
  - Experience of any aspect of the deployment process for operational partnerships between December 2017 and February 2019
  - Working in either WHO Headquarters, Regional Office, Country Office or Cox’s Bazar emergency office between December 2017 and February 2019
- Staff from operational partners
  - Working for one of: GOARN (WHO), RedR Australia, Save the Children UK, Norwegian Refugee Council
  - Directly involved in any aspect of the deployment of workers to Cox’s Bazar between December 2017 and February 2019
- Deployees
  - Workers from one of: GOARN (WHO), RedR Australia, Save the Children UK, Norwegian Refugee Council
  - Deployed to Cox’s Bazar between December 2017 and February 2019 to work within the WHO Cox’s Bazar emergency office
  - No minimum or maximum time limit on deployment during this period

Supplementary Materials 3: Interview Guidance

INTERVIEW GUIDANCE NOTE

Key Informant Interviews – Operational Partnerships Review Cox’s Bazar Rohingya Crisis Response (GOARN/SBP)

Interview Guide for Deployees

- Prior to commencing the interviews, please update the list of key informants with names and designations and interview times as available. Kindly consider how you wish to select persons who have been deployed through GOARN and SBP for their point of view. This will require some advance planning.
- One-on-one interviews of approximately 30-60 minutes are preferred.
- Please take notes throughout the interview and kindly compile this into a brief interview report immediately after. Use common headings to ease analysis later.
- Feel free to probe, asking follow-up questions “What/who/how/examples” to obtain useful observations and recommendations?
- The interview questions below are meant only to guide discussions, feel free to touch on subjects related to this that informants would like to discuss. However, please ensure that the following subjects have been touched on through your discussion on the below, as this will be required to generate recommendations:
  - - - Both GOARN and SBPs should be adequately addressed, if the participant has experience with both
      - Operational aspects (ease of deployment, timeliness/speed of deployment, preparation of candidate by SBP/GOARN/Institution prior to arrival and by WHO at time of arrival, diversity of deployees)
      - Technical contributions (technical areas covered, technical appropriateness of deployed candidate for proposed work (background, experience, skillset), cultural sensitivity, adaptability to working environment)
      - Try to extract concrete examples of successes, constraints and recommendations
      - Establish rapport, explain the intention is to improve these deployments in future operations and assure confidentiality and non-retaliation. Explain how the process of compiling the recommendations will go, addressing the desk review and the key informant interviews. Maintain a neutral attitude.
